# *“No Papers. No Doctor”*: A Qualitative Study of Access to Maternity Care Services for Undocumented Immigrant Women in Denmark

**DOI:** 10.3390/ijerph17186503

**Published:** 2020-09-07

**Authors:** Julia Kadin Funge, Mathilde Christine Boye, Helle Johnsen, Marie Nørredam

**Affiliations:** 1 Midwifery Department, University College of Copenhagen, Sigurdsgade 26, 2200 København N, Denmark; hjoh@sund.ku.dk; 2 Danish Research Centre for Migration, Ethnicity and Health, Department of Public Health, University of Copenhagen, Øster Farimagsgade 5, 1353 København K, Denmark; mcb@sund.ku.dk (M.C.B.); mano@sund.ku.dk (M.N.); 3 Section for Social Medicine, Department of Public Health, University of Copenhagen, Øster Farimagsgade 5, 1353 København K, Denmark; 4 Section for Immigrant Medicine, Department of Infectious Diseases, Hvidovre Hospital, Kettegård Allé 30, 2650 Hvidovre, Denmark

**Keywords:** maternity care, utilization of maternity care, pregnancy, childbirth, undocumented immigrant

## Abstract

The purpose of this study is to explore undocumented immigrant women’s experiences of, as well as their access to, maternity care services during pregnancy in Denmark. Recruiting through the two branches of a non-governmental organization (NGO)-driven health clinic in Denmark, we conducted 21 semi-structured interviews with undocumented immigrant women in Denmark from January 2018 to January 2019. The undocumented immigrant women experienced barriers such as fear of deportation, concerns about payment for services, and uncertainties about rules for access. Many of them described depending on NGO-driven initiatives to access maternity care services and found these as providing a safe environment for care. Our findings contribute insights towards understanding the health behavior of undocumented immigrant women and highlight the need for inclusive care to safeguard the health of the women and their children.

## 1. Introduction

Undocumented migration is defined by the International Organization for Migration as the “movement of persons that takes place outside the laws, regulations, or international agreements governing the entry into or exit from the state of origin, transit, or destination” [1]. The pathways to undocumented migration into European countries can be roughly grouped as entry via the asylum system and eventually having the application rejected, visa-overstaying, and clandestine border crossing including trafficking [2]. An often-overlooked group of undocumented immigrants within the EU are unregistered EU citizens, who are neither employed nor registered as seeking employment in the host country [3]. It is estimated that 1.9–3.8 million undocumented immigrants are living within the borders of the EU [2]. In a Danish setting, the most recent estimate was that 22,900–28,900 undocumented immigrants are living in Denmark [4]. 

In Denmark, undocumented immigrants have restricted access to health care including maternity care [5]. Women with a legal residency in Denmark are entitled to maternity care services free of charge through public hospitals and state-subsidized general practitioners (see Table 1). Undocumented immigrants are solely entitled to healthcare for acute, urgent, or pain-relieving needs [6]. Thus, public maternity care during pregnancy and after giving birth is not accessible for undocumented immigrants [3], and their access to free maternity care services relies on the informal health system.

The Danish Red Cross provides primary health care, including diagnostics for common symptoms and infections, vaccination of children, acute dental care, and maternity care, to undocumented immigrants through a healthcare clinic (hereafter referred to as the Health Clinic) established in collaboration with the Danish Medical Association and The Danish Refugee Council [7]. The Health Clinic opened in 2011, and expanded with a second Danish branch in 2013 and a third in 2019. From its opening until January 2019, the Health Clinic helped 5157 patients, and among those were 757 pregnant women [7].

According to the World Health Organization, being an immigrant in itself serves as a risk factor for having poorer general health than the background population [10]. In Sweden, a study has shown that undocumented immigrants have a higher risk of dying from external causes and circulatory system diseases compared to the Swedish host population [11]. A systematic review concludes that, across European countries, undocumented immigrant women and children have poorer health outcomes than the background population [12] where health risks such as living conditions and pre-migration exposures require specific attention [13]. A Dutch study shows an elevated risk of low birth weight (odds ratio 3.51), premature birth (odds ratio 6.17), and neonatal admissions (odds ratio 1.97) among undocumented immigrants compared to documented immigrants [12]. However, some studies find no difference [14]. The health outcomes and health-seeking behavior of undocumented pregnant women remain understudied areas. Pregnant undocumented immigrants in Europe underutilize maternity care services compared to documented immigrants. They have delayed maternity care during pregnancy care, if any care at all, along with lower screening rates for Hepatitis B, HIV, and syphilis during pregnancy [12,15,16]. Studies conducted in the Nordic countries describe that undocumented immigrant women face barriers to maternity care during pregnancy ranging from lack of legal entitlements to care, feelings of insecurity encountering public health care facilities, poor language skills, and financial difficulties [17,18]. However, the literature is lacking with regards to describing undocumented immigrant women’s experience of accessing maternity care services across sectors. Therefore, this study aims at exploring undocumented immigrant women’s experience of and their access to both formal and informal maternity care services in Denmark.

## 2. Materials and Methods 

The study was a part of an overall research project [19] entailing several studies aiming to provide knowledge about the health and healthcare seeking behavior among undocumented immigrants and their children in Denmark. 

### 2.1. Study Design 

A qualitative research design was chosen for this study, to gain in-depth knowledge of the women’s experiences [20]. Data for this study were collected through 21 individual semi-structured interviews with undocumented immigrant women in Denmark.

### 2.2. Setting and Recruitment

The recruitment site of the study informants was the aforementioned Health Clinic for Undocumented Immigrants. We recruited 21 undocumented immigrant women through purposive sampling [21]. The criteria for inclusion were being pregnant, and attending maternity care at the Health Clinic for Undocumented Immigrants at time of recruitment. The pregnant women were recruited by either the Health Clinic midwife or the first author in the waiting area after a consultation. In total 19 pregnant women were invited. Five of them declined participation due to lack of time and/or finding participation in an interview too risky. For the recruitment of mothers, two different strategies were applied. Firstly, pregnant women attending midwife consultations were asked for permission for the interviewer to contact them postpartum. The women were then contacted by phone 4 months after their due date and an interview was arranged. However, it proved challenging to reach the women who had given consent as many no longer responded to the given phone numbers, and in four cases, the women no longer wished to participate due to lack of time. Therefore, the recruitment strategy was expanded to include the recruitment of new mothers attending the pediatrician or public health nurse at the Health Clinic. Here, three women were asked for an interview by the first author, and two interviews were performed immediately after the consultation. One woman declined an interview and did not give a reason.

### 2.3. Participants

The women interviewed during pregnancy (*n* = 14) were 20–39 weeks pregnant at the time of the interview, and the mothers of newborns (*n* = 7) were interviewed 4–8 months postpartum. The women constituted a heterogeneous group. They were aged between 20 and 42 years old with a mean age of 29.4 and originated from The Philippines, Sudan, Morocco, Pakistan, Kenya, Tanzania, Uganda, and Bosnia. The majority were nulliparous (*n* = 15) and the remaining had 1–3 children. Most of the women lived with their partner, and the partner could be a Danish citizen, have obtained a Danish residence permit, or be undocumented like themselves. The women were employed in the informal sector predominantly as domestic workers, or not be employed at all. Some women were in the process of applying for residency, whereas some of them were neither applying nor planning to apply.

### 2.4. Data Generation

Data were collected from January 2018 to January 2019. The participants could choose where they wanted to be interviewed, and all chose to be interviewed at the Health Clinic. JKF, who is a researcher and a midwife, performed all interviews. For the women who did not speak sufficient English, professional phone translation was provided in their mother tongue. The interviews lasted between 12 and 45 min. 

A semi-structured interview guide was used to generate data [22]. The main questions centred on the experience of being pregnant and being a new mother with no legal residence permit, their thoughts on maintaining their own and their child’s health, and how they experienced encounters with the health system. 

### 2.5. Data Analysis

All interviews, apart from two, were recorded and later transcribed ad verbatim [22]. In two cases, the participants did not feel comfortable with the interview being voice recorded and the recording was therefore replaced by thorough notes taken by the interviewer. In cases where a translator was used during the interviews, only the words of the interviewer and translated participant responses were transcribed and used in the data analysis. The names of women appearing in the article are fictitious, and names of places are anonymized. Nvivo (Alfasoft, Søborg, Denmark) was the analysis software used for coding and organizing the data [20]).

The data was analyzed according to Malterud’s guidelines for systematic text condensation [21,23]. First, all transcriptions were read, and preliminary categories associated with the study aim were formed. Second, we identified all text fragments elucidating the study aim. These meaning units were coded and organized according to the themes developed in the first stage of the analyses process. This de-contextualization allowed for reflection across and within the themes and for an in-depth understanding of the data. Third, this information was condensed and abstracted into groups identifying the themes related to the study aim. Last, the content was synthesized into the main findings [23]. Author JKF undertook all steps of the analysis, but all steps were thoroughly discussed among all authors to enhance the reliability of the analysis. Additionally, findings were discussed with a group of professionals within the area of immigrant health and maternal care.

### 2.6. Ethical Considerations

During the study period, the ethical considerations were discussed among the authors and the management of the Health Clinic as to ensure the protection of our informants. Vigilance was on the precarious legal situation of the participants, and this was taken into careful consideration during the study by ensuring anonymity as well as conducting interviews in safe settings [24]. The participants were recruited after their consultations, to minimize the perception of care being dependent on participation. Furthermore, the participants were made aware that the information given during the interview was confidential and not shared with the staff of the clinic or others.

All participants were given a verbal and written explanation of the study purpose, anonymity, and the right to withdraw participation at any time during the study. Based on this, they all signed consent forms.

Ethical approval was obtained under the University of Copenhagen’s joint record of research projects containing personal data in compliance with the General Data Protection Regulation (Regulation (EU)2016/679). 

## 3. Results

During the process of systematic text condensation, four overarching themes with eleven subthemes emerged (see Table 2).

### 3.1. Access to Public Maternity Care Services

This theme describes which public maternity care services the women used. It describes their expectations about them, as well as their experience of gaining access to maternity care services. 

#### 3.1.1. Experiences with Public Maternity Care Providers

The informants were in contact with public maternity care providers before, during, and after giving birth. When needing care in the early stages of labor, or in relation to the induction of labor, some women experienced that they had to negotiate their entitlement to care with health professionals, making the women feel neither welcomed nor acknowledged. In some cases, they felt they had to argue their case to secure the safety and health of their unborn child.

During the actual birth, the women expressed having felt well treated during encounters with public maternity care providers and that they had received both adequate care and support. They did not feel that their legal status had affected the care they received, nor the staff’s perception of them. Marisol tells: “…They were really nice, and the midwife was really accommodating me well, and the midwife even knew that the hospital asked us to pay, but she said that she is just doing her job. And her job is to take care of me and my baby. She was really, really nice” (Marisol, Int. 18).

For post-natal care, the women experienced being questioned about entitlement to care and about their living conditions. Rahima reports that she felt alienated by these questions: “They asked funny questions, like do we have a home. It’s like they don’t know anything about people who are different from them. We are normal people, just without the normal papers.” (Rahima, Int. 21). At discharge, they were given no appointments for follow-up, apart from one being recommended to seek follow-up for a neonatal infection at the Health Clinic. This caused them to worry and feel insecure. 

#### 3.1.2. Fear of Deportation

Fear of deportation was by far the dominant factor affecting the access to maternity care, as this made the informants hesitant to seek maternity care at public hospitals. Eventually, they all did seek public hospitals, but not until it felt highly necessary, as described by Jessa who had an episode of severe pain during her pregnancy: “I thought I was going to die. It didn’t come abruptly. I felt more and more pain…But I knew we could not go to the hospital. But when I reached the point of thinking it’s hospital or death, we went. I couldn’t be deported if I was dead, you know. Dead people don’t have passports” (Jessa, Int.10).

Even though the women were aware of the hospital staff’s obligation to confidentiality, all contact with a public hospital was seen as posing a risk, which was why the women wished to spend as little time as possible in the hospital during the birth; arriving late and leaving quickly. Despite this attitude, the women clearly expressed that if their child needed hospital admittance, they would adhere to it, no matter what the legal consequence. They feared consequences relating to their reason for staying in Denmark, either being separated from their spouse or losing their job. Applicable for all the women was a profound fear of being separated from their children, as Dina explains: “I am afraid that the police will take me. What if I am not with my daughter at the time, would she have to stay here without me? Or go to Africa alone? It’s horrible to think of” (Dina, Int.14).

#### 3.1.3. Concerns about Payment

The possibility of being required to pay for maternity services served as a barrier for the women, in regards to pregnancy care, but mostly related to birth. Many had heard rumors of other women being charged for services, or the informants themselves had been faced with a payment claim when seeking maternal care. For example, Marisol, who went to the labor ward for an assessment relating to her overdue pregnancy: “...then they checked me and then they checked my tummy. They had ultrasound and checked my water, and said baby is fine, but it’s time to start the birth. What do they call it? Induce the labor. But first we have to pay for my birth” (Marisol, Int.18). Others were faced with the payment claim at discharge or had bills sent to family members or tenants, while some were not. 

The women reported that the cost of giving birth could be between 20,000–25,000 Danish kroner (2600–3300€), which they saw as unmanageable. Most had low incomes, and some were financially dependent on their partner or family members, who also had low incomes, such as social benefits or salaries from low-skilled jobs. Generally, the women expressed that financial hardship was an ongoing concern in their life and that the worries of possibly having to pay for maternity care added to this concern. The women feared that the debt could increase their risk of deportation or affect their chances of attaining residence permits in the future. It also caused worries of whether they would be refused care during birth or be given inadequate care. Furthermore, they worried whether they would end up with unmanageable bills afterwards. However, the concern about payment did not deter any of the women from seeking care during birth, as this was considered unsafe for the baby, nor were any of the women rejected at the labor ward.

### 3.2. Use of Private and NGO-Driven Maternity Care Services

This theme describes the women’s health-seeking behavior when facing barriers to access of the public system. The services included maternity care at the Health Clinic and the use of private maternity care services. Furthermore, the theme describes the women’s experiences of using these services and which barriers they faced.

#### 3.2.1. Feeling Safe at the Health Clinic

The undocumented immigrant women expressed feeling deeply grateful to the Health Clinic for providing a place to seek maternity care. They were aware that the clinic staff were volunteers and worked for altruistic reasons, and it was essential for the women to know that the purpose of the clinic was to provide healthcare for undocumented immigrants. The women felt safe in there, and they felt safe in knowing that they would not be reported to the authorities. The staff did not pay attention to the lack of residence permits during visits, and as Christina expresses below, it made her feel welcomed and safe: “Honestly, they are so friendly here, and it’s so nice to see that people really want to help you, even though you are foreign…and even though you don’t have residency. You don’t have nothing, but you have the clinic. And know that they won’t report you to the police. It feels very safe” (Christina, Int. 7). 

#### 3.2.2. Challenged by Logistics

The availability of free healthcare services for undocumented immigrants was limited to the two largest Danish cities during the study period. This gave some of the women long transportation hours to reach the clinics. Dina told that she had logistic obstacles to reach the clinic: “I want to come as the midwife has told me, but sometimes it is just not possible. It is too far and costs too much. I can’t do anything about it. I have to come some other time then” (Dina, Int.14).

Another logistic obstacle was the limited opening hours at the clinics, as they only offered midwifery consultations once a week. It was challenging to get off work, both for the women and their partners or other networks whom they depended on for transport, translation, or emotional support. There were also women who either lived near the clinics or simply considered transport and waiting time at the clinic as a premise in their situation: “...no, its fine. I take the train. Sometimes two. I know the staff are volunteers, and I am just grateful that they want to help pregnant women like me” (Aisha, Int.8).

Some of the women did not live close to a hospital, did not have any means of transportation, or lived where public transport was not very efficient, especially at odd hours. This posed a challenge when needing to reach a hospital for the birth. In these cases, the women had to get help from their network, as taking a taxi was considered too expensive. 

#### 3.2.3. Paying for Private Healthcare Services

Some of the women used private healthcare services during their pregnancy. One of these private services was an ultrasound scan of the fetus in private clinics. The women perceived ultrasound scans as necessary, and having ultrasound scans was perceived as being a part of a normal pregnancy. It was important for them to both connect with the fetus to make sure that there were no problems with it. Therefore, most women went to see the gender of the baby, or in early pregnancy to see the baby for emotional reasons. A few women went because there was uncertainty regarding the due date and they wanted an assessment through the ultrasound scan. 

Some of the women perceived the price of ultrasound scans as high (approximately 70 euros), but despite their limited financial means they found the expenditure worth prioritizing. Others did not consider the cost as particularly high, as they were aware that costs were connected to it both for Danish citizens as well as for themselves in their countries of origin. 

In addition to paying for an ultrasound scan, some women were also seen by a general practitioner. One woman reported that she went to see her husband’s general practitioner in early pregnancy because she did not know where else to go. She was asked to pay for the consultation, and therefore only made a single visit. Another woman was rejected completely due to her lack of a residence permit: “We asked my husband’s doctor, but he said: No! No papers. No doctor” (Mariam, Int.13).

### 3.3. Perception of Entitlements to Care

This theme describes the women’s perception of their entitlements to care, exploring their wishes for fair treatment, and the uncertainty they experience regarding care.

#### 3.3.1. Wishing for Fair Treatment

The limited access to maternity care was perceived as unfair. The pregnancy and a deep concern for the baby’s health gave the women a strong perception of them having the right to care. “When I don’t have papers. I feel sad about that. Why should my child not get the same help as other children? A child is a child” (Mariam, Int. 13). As Dina expressed, the perception of entitlement was supported by a profound notion of fairness and a wish to be treated equally to others: “…but if the midwife in at the hospital says, “You are not Danish. you can go home or pay” I will think: I am not Danish, but I need help with my baby. I am a human being” (Dina, Int.14). Even though being pregnant or having a small child played a role in the perceptions of entitlement, the women were aware that it would not improve their chances of being granted residency. They were also aware of the fact that their children would only obtain a residence permit based on the legal status of their parents. While they were aware that their stay in Denmark was not legal, they did not see themselves as criminals or as doing anything wrong. Several expressed that their stay in Denmark was based on an urgent need. This factor supported their perception of entitlement. However, in their encounters with the public healthcare system, both the limited access and questioning of the women’s entitlement caused them to feel like criminals, which did not correspond with how they saw themselves.

There was a shared notion among the informants that if a staff member liked women from a certain country, or was sympathetic to the woman’s situation, the access to and quality of care would improve. Thereby, the granting of access was seen as being up to the individual healthcare professional, more so than rules and legal rights. For some women, the perception of entitlement was affected by comparison to the level of access in their country of origin. They stressed that in their countries of origin, there was a high level of co-payment and corruption within the healthcare system. This comparison made them feel that the rules in Denmark were fair, even though the rules did place hardship and worries on the women. Another factor affecting the women’s perception of entitlement was the severity and urgency of their need for maternity care. Even though they were not all confident that they would receive care, they felt entitled to care both during birth and in cases such as severe pain or bleeding. In contrast, they did not always feel entitled to preventive maternity care.

#### 3.3.2. Uncertainty of What to Expect

Uncertainty played a marked role in the women’s lives and was rooted in several issues. There was uncertainty related to the possibilities of obtaining a residence permit and thereby gaining rights to maternity care. This issue was present in the life of the women who were either involved in an application process or had some sort of prospect of obtaining a residence permit. One woman described it as “an exhausting waiting game” and talked about how she had been involved in the application process for years. This uncertainty was closely linked not only to the fear of never obtaining the permit, but also to whether they would have continually limited access to maternity care during birth and whether this would affect the health of their newborns.

Another area where the women were affected by uncertainty was regarding care during birth. Some reported that they were uncertain about where to go, and more so talked about distress regarding whether they would be allowed care during birth and whether the quality of care would be affected by the lack of a residence permit. One woman expressed that she had never had previous contact with the hospital and had heard negative stories about other undocumented immigrants who had not been treated well. This made it feel like a gamble to plan to give birth at the hospital.

The perception of a varying administration of access to public maternity care led to some uncertainty. Rosa expressed a feeling of unfairness: “I think they should be more precise about it, because I know some people in the same situation like me, and they have access to hospital, and things like that, but in another region. But in my region, it’s like they have other rules. So, it’s like kind of unfair” (Rosa, Int.1).

### 3.4. Being Dependent

This theme describes how the women depend on help, both from the Health Clinic and their social network. The theme also includes what sources of information they turn to for support and information regarding pregnancy and birth.

#### 3.4.1. Dependency on the Health Clinic

The Health Clinic was the women’s preferred place to seek maternity care, and for most it was the only place to seek care during pregnancy. When they did not have anywhere else to go, they felt dependent on the help they received at the clinic: “...I solely depend on this place. If I could not go here, where would I go?” (Rahima, Int.21). The informants not only felt dependent in regard to the care they received at the clinic, but also expressed that they needed the clinic to establish contact with the hospital at the time of the birth: “The clinic can assure me that nothing will happen to me when I go there. Because I remember before I got pregnant, I had to do some x-rays, and that was a bit risky, you know, going to the hospital, but the clinic assured me that nothing would happen. They made an appointment with the doctors (at the hospital), and therefore I got treatment and they were nice to me. I would not go without a referral from the clinic” (Milena, Int.6).

This dependency was also expressed by worries about having a medically urgent need, such as bleeding or going into labor, outside the clinic’s opening hours. The women did not have a strategy for such a situation, but simply expressed despair when asked about it. When Mabel was asked where she would seek care, if needed, outside the Health Clinic opening hours, she replied: “That’s what I am afraid of! Where can I go? I simply don’t know. I have nowhere else to go. I would just have to wait then” (Mabel, Int.2).

#### 3.4.2. Seeking Information

When having questions regarding pregnancy issues, none of the women turned to official sources such as maternity and emergency services for advice. Rather, they turned to friends and family who had either professional experience with maternity care or personal experience of their own. Asking family members was a way of obtaining both information and comfort. However, as these family members were living in the women’s country of origin, the cost of telephone calls limited contact to some extent.

Another used source of information was the internet, not only to get answers to immediately raised questions but also for obtaining knowledge regarding birth and breastfeeding: “I attended antenatal class in YouTube. Only because I don’t know how else I would prepare in Denmark. Especially if you don’t have social security number, so it was like self-study” (Rosa, Int.1). The internet was seen as an easily accessible source of information. However, the women were aware that the internet was not always a reliable source and that information should be read with precaution.

#### 3.4.3. Support from Social Networks

The women who had partners with Danish residence permits experienced a deep dependence on these men. “Well, it’s a little bit hard when you come to a different county, different town, when you don’t have nobody, so I only depend on my husband” (Christina, Int.7). For most informants, the partners were responsible for establishing the initial contact with the Health Clinic and hospitals. During consultations and hospital admittance, the partner, in some cases, served as a linguistic translator. The women also experienced their partners as their “advocates”, making sure that their needs were met by healthcare professionals, but also in supporting them in dealing with the legal aspects. There were other aspects where the women expressed a need for protection. Mabel reported that she only left the house in the evenings because her husband could then go with her. In those instances, she felt safe, as he would be able to speak to the police if they were stopped, and be able to speak Danish to not draw unnecessary attention: “…I never leave the house without him (the husband). I need him for protection. What if the police stop me? Maybe they won’t think about me, when he can answer in Danish” (Mabel, Int.2).

Even though most of the women expressed that they had a limited social network and that separation from family and other social networks in the country of origin was mentioned as a stressor, friends were the key to maternal health services. This was such because the friends knew about ways to obtain maternity care as an undocumented immigrant or they could offer support in terms of providing knowledge about health and provide practical aid such as transport, linguistic translation, or emotional support.

## 4. Discussion

This study examined how undocumented pregnant women experience maternity care services during pregnancy as well as their access to public maternity care in Denmark. Our analyses revealed that they encountered several barriers to access the public health system and thus relied on alternative strategies such as using NGO clinics and informal networks. We will discuss our findings of health-care seeking behavior in relation to groups of undocumented immigrant women in other countries, as well as the potential consequences for birth outcomes. 

### 4.1. Barriers to Public Maternity Care Services

In this study, we found that one of the main barriers to accessing public maternity care was the fear of being reported to the migration authorities when in contact with public health care providers. Another barrier was concern about payment and the consequences of not being able to pay. Scandinavian studies among undocumented immigrant women support these findings, and point to the continuous insecurities having both psychological and physiological impacts on their pregnancies [25,26]. Maternal well-being and stress during pregnancy can impact fetal development and pregnancy outcome [10]. After the data collection of the present study was finalized, a change to the Danish Health Care Act was adopted stating that emergency care could no longer be provided free of charge [27]. Undocumented immigrant women are allowed to give birth at Danish hospitals. However, they can be charged for services related to delivery afterwards. This change in law could potentially result in them refraining from seeking necessary emergency care. In this study, we also found that the undocumented immigrant women experience that restrictive practices regarding entitlements vary across the Danish hospitals as well as among health providers. A Danish study describes that nurses experience feeling insecure about the correct standard procedure when treating undocumented immigrants in emergency room admissions [28]. Our study highlights the need for clear guidelines and health providers who are well-informed in relation to entitlements. Furthermore, our results indicate that increased awareness among public maternity care providers concerning the experiences of undocumented immigrants is needed. Most importantly, it highlights that health provision and immigration procedures should be delinked for the safety of undocumented immigrant women and protection of health providers. This is the case in 11 European countries where undocumented immigrant children, and in some cases pregnant women, can access health care services on equal terms with the host population [29].

### 4.2. Accessibility of Maternity Care Services in the Informal Sector

The analysis revealed that the undocumented pregnant women in this study felt safe at the Health Clinic, and that they established a trust-based relationship with the health providers volunteering there. Studies have shown, that a caring relationship between providers and the undocumented immigrant women is essential to facilitate access to quality maternity care during pregnancy [10,25,30]. When health providers have cultural insights and effective communication skills, the women can feel in control of the encounter [30], which is crucial as findings in this present Danish setting indicate that access to public health care is dominated by feelings of insecurity and mistrust. In our study, we found that the undocumented pregnant women could feel dependent on the availability of the Health Clinic for health services, as well as feeling dependent on their network and especially their spouse for information and protection. The importance of support from health care professionals and networks is also highlighted by Balaam et al., as being important to facilitate care in the transition to becoming a mother [30], and thus puts the woman in a vulnerable position. The de-facto availability of health services at the Health Clinic is challenged. The Health Clinic offers midwifery consultations once a week and the women have long transportation hours and costs related to this. A WHO report supports that logistic barriers are important for optimal quality of care [10]. All the women interviewed for this present study managed to attend the Health Clinic at least once during pregnancy, which is significantly less than the WHO recommendations for optimal antenatal care [10]. However, there is a group of undocumented immigrant women who do not manage to attend the Health Clinic at all because they live too far away. In addition, they do not know where, or do not dare, to seek help for acute conditions occurring outside the clinic’s opening hours, which demonstrates a clear unmet need for accessible health services.

Several of the women in this study paid for ultrasound scans conducted by private providers. They perceived this as being part of what they called a “normal pregnancy”, and the price was perceived as either manageable and/or inevitable. The WHO only recommends one ultrasound scan before gestational week 24 [31] and the Danish Health Authorities recommend two ultrasound scans during pregnancy [9] in order to estimate gestational age and detect fetal anomalies. According to these guidelines, ultrasound scans are also important for women to know when to seek timely maternity care, as they provide them with information to perform their right to abortion before the legal limit of 12 gestational weeks in Denmark. The fact that the undocumented pregnant women in the present study were required to pay for an ultrasonic scan could result in unequal opportunities for optimal care. This could leave the women without financial capabilities, as well as their children, in a vulnerable situation which excludes them from crucial information about the health and well-being of their child. 

### 4.3. Consequences of Delayed Care-Seeking

The analysis revealed that the undocumented immigrant women experienced several barriers such as fear of deportation and concern about payment when accessing maternity care services. These factors can lead to underutilization of maternity care services, or no utilization at all. Studies among immigrant groups in general show a tendency of underutilizing maternity care services during pregnancy [32], thus our findings may not be surprising. Several studies of maternal and newborn health conducted among documented immigrants point at various reasons for delayed care-seeking leading to adverse birth outcomes such as maternal mortality and stillbirths [33,34,35]. From a patient perspective, the authors of an audit study propose that a lack of symptom awareness, refusal, or hesitation to seek care [33], as well as language barriers [34] all play a role. From a provider perspective, suboptimal quality of care, misdiagnosis, insufficiently staffed facilities, and lack of clinical guidelines affect adverse birth outcomes [34,35]. It is reasonable to assume that these findings are also applicable to undocumented immigrants, leaving this group at excessive risk of adverse outcomes when they experience several barriers to accessing care. In the United States, maternal and newborn health improved significantly when allowing pregnant undocumented immigrant women access to free antenatal care services [36]. The authors find that increased utilization and quality of care decreases child mortality within the first year of life and lowers the risk of extremely low birth weight. These findings, along with our study, highlight the need for, and benefits of, improving access to maternity care services for undocumented immigrants in Denmark. 

### 4.4. Methodological Strengths and Limitations

A strength of this study was the heterogeneity of the study population; we were able to recruit women of different ages, country of origin and living circumstances. Furthermore, given the difficulties in recruiting undocumented immigrants for research in general [37], it is considered a strength of this study that we succeeded in recruiting 21 women. 

Information regarding migration history, lifestyle, and general health including mental health would have added to the analysis and given valuable perspectives to the research question and possibly added to the heterogeneity of the participants. However, in respect of the Health Clinic’s policy on safeguarding the safety of their patients and due to the time aspect of the data collection, these issues were not included in the data collection. 

The different entitlements to care for undocumented immigrants across Western countries affects the transferability of the study. However, a number of findings are confirmed in similar settings in other countries [25,26]. It can be assumed that the issues raised in this study are applicable for pregnant undocumented immigrants living in countries with similar legislation. Informants were recruited through the Health Clinic, and they had not allocated time to be interviewed beforehand, thus impacting length and level of detail in their answers. Our informants were utilizing maternity care services to some extent, and it is liable that they experience access to maternity care in general differently than women who did not seek care at the Health Clinic. Furthermore, the Health Clinic provide services free of charge that they cannot access elsewhere, which can lead to biased answers towards painting a more positive picture of the Health Clinic due to the gratitude they felt. Lastly, two of the informants asked for the interview not to be recorded, and notes were taken. Thus, the notes taken might lack nuances and accurate information [20,22].

## 5. Conclusions

Undocumented pregnant women experience several barriers to accessing maternity care services in Denmark. Accessing public maternity care services is, apart from legal restrictions, dominated by fear of being reported to the immigration authorities and hence deportation, concerns about payment, and the management of rules from the health provider’s perspective. Many of them depend on NGO-driven initiatives, the Health Clinic, and their personal networks for care and protection. The undocumented immigrant women experience feeling safe in accessing maternity care services at the Health Clinic. However, some also have substantial logistic barriers to attending. 

Our findings contribute insights towards understanding health-seeking behavior among undocumented pregnant women and highlight the need for accessible and quality maternity care services. To safeguard the health of the women and their children, inclusive policies for access to health care in Denmark for all must be implemented, as well as heightened awareness from public health care providers to provide care to this group. Further studies are needed on the health needs and health-seeking behavior of women not in contact with the Health Clinic, to fully understand the challenges of access to maternity care for undocumented pregnant women.

## Figures and Tables

**Table 1:** Public maternity care services in Denmark and maternity care services provided at the Health Clinic.

**Public maternity care services in Denmark**
Maternity care services are provided according to the Danish Health Authority’s national recommendations. The regional health authorities are responsible for managing and implementing the services to meet the scope within the provided recommendations [8]. The offered maternity care services during pregnancy when having a low-risk pregnancy [9] include a first visit to a general practitioner who refers them to antenatal visits and delivery at a public hospital. Provision of maternity care services after giving birth differs across regional health authorities. Whether they stay at a maternity ward depends on the mother’s parity and risk as well as the ward’s capacity. However, there will always be follow-up via either telephone consultation or home visit.
**Maternity care services provided at the Health Clinic**
Undocumented immigrant women can access free maternity care services at the Health Clinic [7]. Maternity care services during pregnancy are provided by one of the Health Clinic’s midwives once a week. The midwifery care at the Health Clinic includes urine test for proteinuria and glucose, measurement of blood pressure, blood test for Human Immunodeficiency Virus, Hepatitis B and blood glucose levels, auscultation of fetal heart rate, abdominal examination to assess fetal position and size. These are services similar to the tasks provided by the general practitioner and midwife in the authorized health system [9]. At the end of each consultation, timing of the next visit is decided. It is not possible to have routine ultrasonic scans at the Health Clinic.

**Table 2:** Themes and subthemes of the study.

Themes	Subthemes
1. Access to public maternity care services	Experiences with public maternity care providers Fear of deportation Concerns about payment
2. Use of private and non-governmental maternity care services	Feeling safe at the Red Cross Health Clinic Challenged by logistics Paying for private healthcare services
3. Perception of entitlements to care	Wishing for fair treatment Uncertainty of what to expect
4. Being dependent	Dependency on the Red Cross Support from social networks Seeking information
